# Geographical Variation in SARS-CoV-2 Transmission Potential in Massachusetts

**DOI:** 10.3390/epidemiologia7010015

**Published:** 2026-01-21

**Authors:** Ina Sze-Ting Lee, Xinyi Hua, Jing Xiong Kersey, Kayoko Shioda, Gerardo Chowell, Isaac Chun-Hai Fung

**Affiliations:** 1 School of Public Health, Boston University, Boston, MA 02118, USA; inaleect@bu.edu; 2 Department of Microbiology, Immunology and Molecular Genetics, College of Medicine, University of Kentucky, Lexington, KY 40536, USA; xinyi.hua@uky.edu; 3 Department of Biostatistics, Epidemiology and Environmental Health Sciences, Jiann-Ping Hsu College of Public Health, Georgia Southern University, Statesboro, GA 30460, USA; jxkersey@georgiasouthern.edu; 4 Department of Global Health, School of Public Health, Boston University, Boston, MA 02118, USA; kshioda@bu.edu; 5 Center on Emerging Infectious Diseases, Boston University, Boston, MA 02118, USA; 6 Department of Population Health Sciences, School of Public Health, Georgia State University, Atlanta, GA 30303, USA; gchowell@gsu.edu

**Keywords:** COVID-19, epidemiology, non-pharmaceutical interventions, reproduction number, time series analysis, United States

## Abstract

Background/Objectives: This ecological study aimed to investigate changes in the time-varying reproduction number (Rt) of SARS-CoV-2 across six regions of Massachusetts from 2020 to 2022 and to evaluate the impact of various nonpharmaceutical interventions (NPIs) implemented in 2020 by examining associated changes in the Rt. Methods: COVID-19 incident case data from the Johns Hopkins University database were adjusted for reporting delays using deconvolution and for underreporting via a Poisson-distributed multiplier of 4. Negative and zero counts were corrected using imputation. Rt was estimated using R package *EpiEstim* (Version 2.2-4) with a 7-day sliding window from 2020 to 2022 and with non-overlapping time windows between policy changes in 2020. Results: From 2020 to 2022, Massachusetts experienced five COVID-19 surges, linked to the wild-type strain and emerging variants, with Rt exceeding 1 during each wave and stabilizing at or dropping below 1 during low-incidence phases. School closure and gathering restrictions, the first major intervention, were associated with a 14.7% statewide reduction in Rt (95% credible interval (CrI): −23.6%, −5.6%), with greater reductions in high-density areas such as Boston (−16.9%; 95% CrI: −26.9%, −7.5%). No statistically significant changes in Rt were found to be associated with other NPIs in 2020, including the mask mandate, reopening phases, travel restrictions and quarantine requirements, and curfews. Conclusions: Our findings highlight the different NPIs’ varying impacts on COVID-19 transmission dynamics across regions in Massachusetts in 2020 and underscore the importance of early interventions for future pandemic preparedness.

## 1. Introduction

Severe acute respiratory syndrome coronavirus 2 (SARS-CoV-2) emerged as a global health crisis, prompting widespread public health interventions to reduce transmission. Massachusetts serves as a valuable case study for understanding pandemic dynamics due to its robust healthcare infrastructure, which facilitates in-depth analysis of coronavirus disease 2019 (COVID-19) transmission and epidemiological patterns. The state’s top-ranked healthcare access and favorable social determinants of health provide important context for examining viral spread and control [1], particularly in relation to non-pharmaceutical interventions (NPIs) and healthcare resource utilization. Additionally, Massachusetts was the site of one of the earliest major clusters, linked to a superspreading event at an international business conference in Boston [2], highlighting the complexities of early outbreak dynamics.

The time-varying reproduction number (Rt) represents the expected number of secondary cases generated by an infected individual at a given time [3]. When Rt > 1, an epidemic grows; when Rt < 1, an epidemic subsides. As Rt is relatively simple to understand, it serves as a critical metric for assessing disease transmission dynamics over time. Rt is influenced by both pathogen-specific characteristics and external factors such as human mobility, population immunity, public compliance with interventions, and healthcare accessibility [3]. Monitoring Rt enables the evaluation of NPIs, informs epidemic modeling, and facilitates the strategic adaptation of response measures during outbreaks. Our prior research has investigated the temporal variation in the Rt of COVID-19 transmission at the state and/or sub-state levels in parts of the United States [4,5,6,7,8,9,10,11,12]. Here, we extended our research to the state of Massachusetts.

This study examines the transmission dynamics of SARS-CoV-2 in Massachusetts, with the following objectives: (1) to analyze variation in SARS-CoV-2 transmission across the six regions of Massachusetts from January 2020 to July 2022; and (2) to evaluate the association between NPIs and Rt in these regions in 2020 (with a county-level sensitivity analysis). This study aims to provide insights into the differential impact of the pandemic across the state.

## 2. Materials and Methods

### 2.1. Data Acquisition

Data were obtained from the COVID-19 Unified Dataset compiled by Johns Hopkins University Center for Systems Science and Engineering, available through their GitHub repository ‘CSSEGISandData’ [13,14]. This dataset includes daily confirmed incident case counts, defined as positive polymerase chain reaction (PCR) test results, reported at the state and county levels from 22 January 2020 to 9 March 2023. County-level population data were sourced from the American Community Survey conducted by the United States Census Bureau [15,16]. In addition, a review of the Governor’s Orders issued by the Commonwealth of Massachusetts was conducted to document the timing of NPI implementations and subsequent relaxations (Table A1).

### 2.2. Statistical Analysis

#### 2.2.1. Unit of Analysis

**Figure 1:**
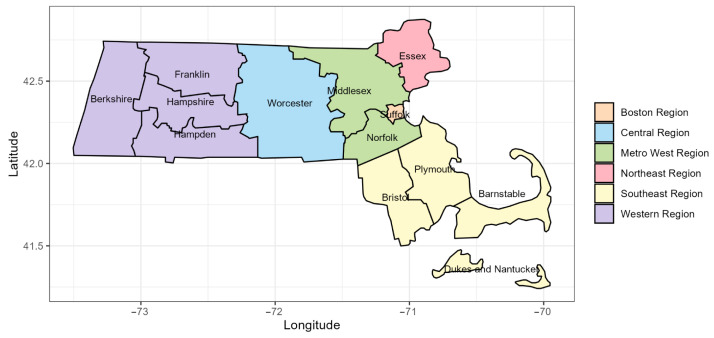
Grouping of the 14 Massachusetts counties by region: Boston (Suffolk); Central (Worcester); Metro West (Middlesex and Norfolk); Northeast (Essex); Southeast (Barnstable, Bristol, Dukes, Nantucket, and Plymouth); and Western (Berkshire, Franklin, Hampden, and Hampshire).

In this study, the 14 counties of Massachusetts were grouped into six regions based on county boundaries. Specifically, the Boston region includes Suffolk County; the Central region includes Worcester County; the Metro West region includes Middlesex and Norfolk counties; the Northeast region includes Essex County; the Southeast region includes Barnstable, Bristol, Dukes, Nantucket, and Plymouth counties; and the Western region includes Berkshire, Franklin, Hampden, and Hampshire counties (Figure 1).

To assess whether regional aggregation obscured within-region heterogeneity in transmission dynamics or policy impacts, we conducted a county-level sensitivity analysis. All steps from the previous sections were repeated using individual counties as the unit of analysis (13 in total, with Dukes and Nantucket aggregated in the original dataset). County-level Rt estimates and percentage changes across policy periods were then compared with region-level results to evaluate robustness.

#### 2.2.2. Data Cleaning, Deconvolution and Multiplier

To estimate Rt, negative incident case counts were first corrected. In addition, adjustments were made to the weekend effect, in which no cases are reported on weekends, to reduce artificial spikes in Rt. Negative values were identified and imputed using a local average of data points from three days before to three days after the corresponding date, resulting in a total imputation window of seven days. Zero values reported during weekends and holidays were imputed using the same method. For data preceding 6 March 2020, which was chosen to align with the beginning of significant COVID-19 case reporting in Massachusetts, zero case counts were not imputed in order to preserve the original data. Imputation near the end of the dataset was performed by averaging available surrounding values, accounting for boundary conditions. These methods were consistently applied to county-level data from all counties in Massachusetts, ensuring uniform corrections and enabling reliable analysis of infection trends and Rt estimation.

Using the R package *incidental* [17], deconvolution was performed on the observed incident case count data to estimate the date of infection by adjusting for the delay between infection and case reporting. This adjustment used the package’s default distribution for COVID-19, which is based on a Florida dataset from 2020 [17]. The *incidental* package employs an empirical Bayes method to fit a regularized likelihood model on a spline basis, thereby removing noise and smoothing the deconvoluted incident case count curve. In the *incidental* package, the parameter *dof_grid* specifies an integer vector of degrees of freedom for the spline basis used in the deconvolution model. We expanded the default range to include values from 6 to 40 in increments of 2, to increase flexibility in model fitting and explore sensitivity to different spline configurations. To estimate daily infection counts, 1000 deconvoluted time series of incident cases were generated. A Poisson-distributed multiplier of 4 was then applied to each time series to account for underreporting of asymptomatic and mildly symptomatic infections [6,18], producing a total of 10,000 time series of estimated daily incident infection counts. The median and 95% credible interval (CrI) were then derived from these 10,000 time series. Additional sensitivity analyses were performed using multipliers of 3.4 and 4.7, corresponding to the United States CDC’s estimated lower and upper bounds for the number of infections per reported case [19].

#### 2.2.3. Rt Estimation

Rt was estimated from the daily incident infection counts using the instantaneous reproduction number method proposed by Cori et al. [3], implemented using the R package *EpiEstim*. Rt is defined as the ratio of the number of new cases at a given time to the total infectiousness of infected individuals at that time. An Rt value greater than 1 indicates that the infection is spreading, while a value less than 1 indicates a decline in transmission.

In *EpiEstim*, the prior distribution for the reproduction number Rt is specified using two parameters: *mean_prior*, which defines the mean of the prior distribution, and *std_prior*, which defines its standard deviation. The default values for both are 5. For this analysis, we adopted more conservative assumptions by setting *mean_prior = 2* and *std_prior = 2*. These adjustments were incorporated into the estimation of Rt across each time window corresponding to the implementation or relaxation of NPIs in Massachusetts.

The 7-day sliding window Rt analysis covered the period from 22 January 2020, which marks the first date in the COVID-19 Unified Dataset, to 8 July 2022, the final day before Massachusetts transitioned from daily to weekly COVID-19 reporting. For the main analysis, Omicron-specific serial interval parameters were used, with a mean of 2.9 days and a standard deviation of 1.64 days [20]. A sensitivity analysis using statewide-level data was conducted with an alternative serial interval distribution based on early pandemic data, which had a mean of 4.6 days and a standard deviation of 5.55 days [9,21]. A sensitivity analysis was also conducted using the default prior distribution for Rt (mean, 5; serial interval, 5). For each Rt estimate, 10 values were sampled from the posterior distribution. From the 10,000 time series of estimated daily infection counts, a total of 100,000 time series of Rt estimates were generated, from which the median and 95% CrI were calculated.

The policy change Rt analysis covered the period from 22 January to 31 December 2020. Seven non-overlapping time windows were used, aligned with specific dates corresponding to Massachusetts Executive Orders related to the implementation or relaxation of NPIs (Table 1). These NPIs were selected because they directly affected social interactions and mobility, were uniformly implemented statewide, and were most relevant for evaluating SARS-CoV-2 transmission dynamics at the population level. Other government orders (Table A1), such as those concerning licensing, insurance, or health care operations, were excluded as they primarily applied to specific sectors and were less directly related to community-wide transmission. Each time window represents a combination of NPIs in place as prior NPIs may continue to be implemented. For each time window, median Rt estimates and their 95% CrI were calculated. The effect of these policy changes on transmission dynamics was assessed by calculating the percentage change in Rt between successive time windows throughout 2020. The median and 95% CrI of the percentage change were obtained via bootstrapping. A simple linear regression analysis was conducted to examine the relationship between percentage change in Rt and the log_10_-transformed population density of each region.

#### 2.2.4. Statistical Language

All statistical analyses were performed using R version 4.4.1 (R Core Team, R Foundation for Statistical Computing, Vienna, Austria, 2024). Important R packages used in this paper include the *incidental* package (version 0.1) for deconvolution and *EpiEstim* package (version 2.2.4) for Rt estimation. The R script for this project is provided in Supplementary File S1. Our custom functions can be found in Supplementary File S2.

## 3. Results

**Figure 2:**
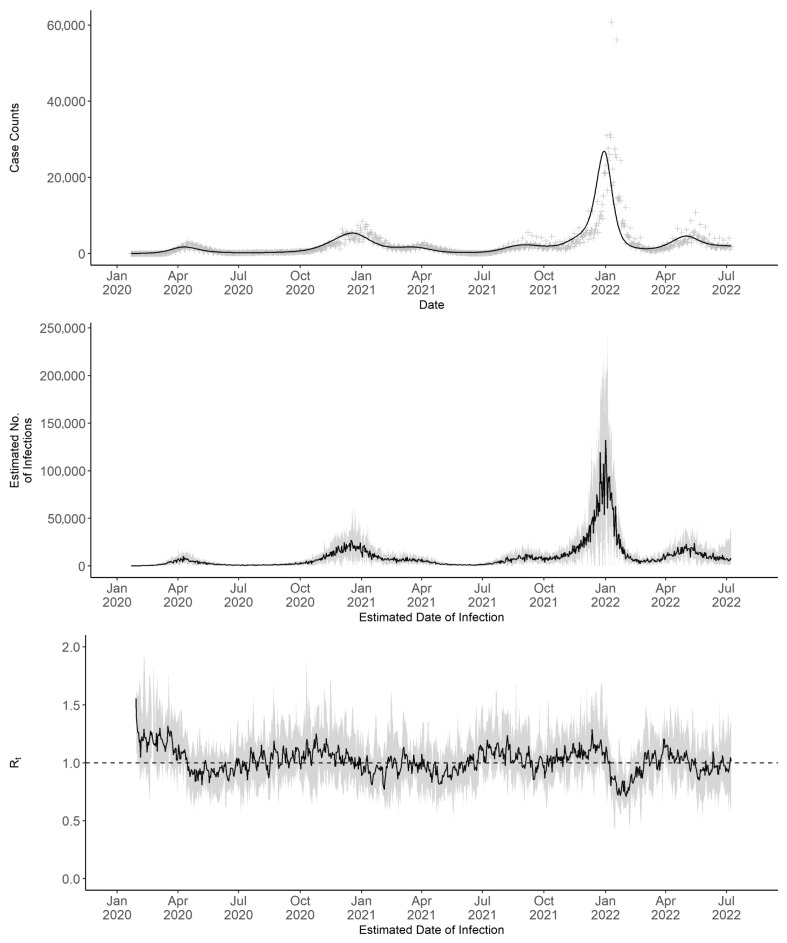
Daily observed incident case counts, deconvoluted incident case counts, estimated incident infection counts, and 7-day sliding window Rt in Massachusetts from 22 January 2020 to 8 July 2022. Observed daily incident case counts, by report date, are displayed as grey points, and deconvoluted daily incident case counts, by estimated infection date, as a black line in the upper panel. The median estimated daily number of new infections (black line) with corresponding 95% CrI (grey area) is shown in the middle panel. The median 7-day sliding window Rt (black line) with corresponding 95% CrI (grey area) is shown in the lower panel.

In early 2020, incident case counts and estimated infections remained low, indicating minimal transmission (Figure 2). This was followed by a gradual increase through mid-2020, marking the onset of periodic fluctuations. These fluctuations continued throughout 2021, with observable peaks in December 2020 and September 2021 indicating elevated case counts; however, neither peak approached the pronounced surge recorded in early 2022 during the Omicron wave. Following this surge, cases declined rapidly and then stabilized, with relatively minor fluctuations through mid-2022.

### 3.1. 7-Day Sliding Window Rt

The 7-day sliding window Rt fluctuated throughout the COVID-19 pandemic in Massachusetts (Figure 2). In early 2020, Rt remained above 1, indicating sustained transmission. By mid-2020, Rt declined. During the summer and fall of 2020, it fluctuated around 1, suggesting near-equilibrium transmission levels. Increases in Rt were observed in October and November 2020. By late 2020, Rt again exceeded 1. After fluctuating near or below 1 for several months, Rt increased during the summer and in November 2021. The November peak was followed by a surge in cases and estimated infections in December. Following this surge, Rt declined and remained near or below 1 through mid-2022.

**Figure A1:**
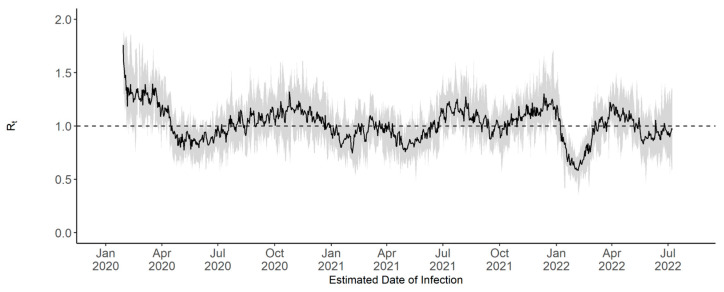
Sensitivity analysis of the 7-day sliding window Rt in Massachusetts from 22 January 2020 to 8 July 2022. The Rt was estimated using an alternative serial interval distribution derived from early pandemic data, with a mean of 4.6 days and a standard deviation of 5.55 days. The prior distribution of Rt was specified with a mean of 2 and a standard deviation of 2. The median 7-day sliding window Rt is represented by the black line, with 95% CrI shaded in grey.

**Figure A2:**
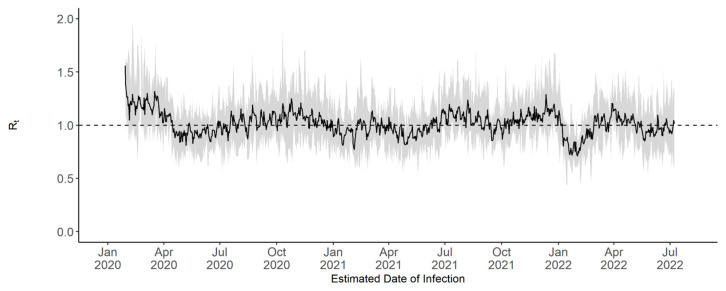
Sensitivity analysis of the 7-day sliding window Rt in Massachusetts from 22 January 2020 to 8 July 2022. The Rt was estimated using the Omicron-specific serial interval distribution, as in the main analysis, with a mean of 2.9 days and a standard deviation of 1.64 days. The prior distribution of Rt was specified with a mean of 5 and a standard deviation of 5. The median 7-day sliding window Rt is represented by the black line, with 95% CrI shaded in grey.

**Figure A3:**
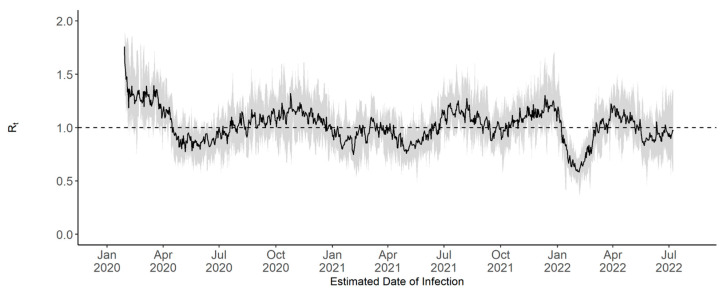
Sensitivity analysis of the 7-day sliding window Rt in Massachusetts from 22 January 2020 to 8 July 2022. The Rt was estimated using an alternative serial interval distribution derived from early pandemic data, with a mean of 4.6 days and a standard deviation of 5.55 days. The prior distribution of Rt was specified with a mean of 5 and a standard deviation of 5. The median 7-day sliding window Rt is represented by the black line, with 95% CrI shaded in grey.

**Figure A4:**
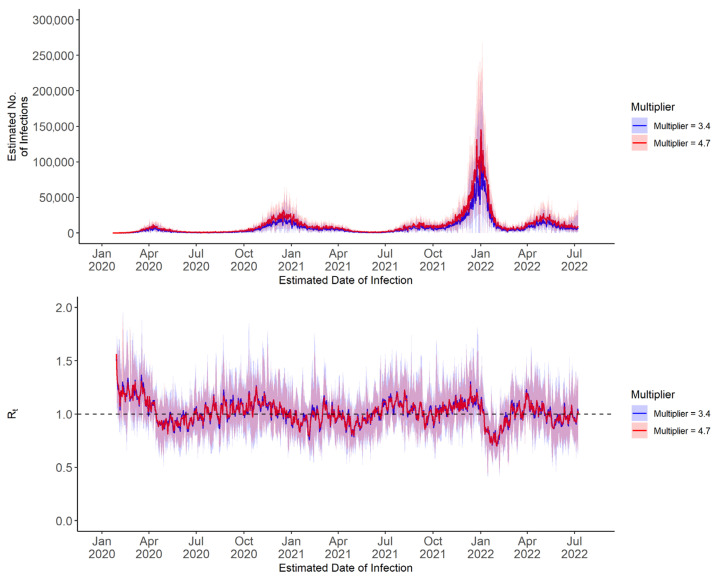
Sensitivity analysis of estimated infection counts and 7-day sliding window Rt with corresponding 95% CrI for Massachusetts statewide from 22 January 2020 to 8 July 2022, using two infection multipliers (3.4 in blue, 4.7 in red). The upper panel shows the median estimated number of new infections with the corresponding 95% CrI. The lower panel shows the median 7-day sliding window Rt with the corresponding 95% CrI.

Sensitivity analyses were conducted to assess the effect of using an alternative serial interval distribution on Rt estimates. This distribution, with a mean of 4.6 days and a standard deviation of 5.55 days, was derived from early pandemic data [9,21]. These parameters were applied to the statewide 7-day sliding window Rt estimation (Figure A1). Additional analyses tested other combinations of serial interval and prior parameters (Figure A2 and Figure A3). Results showed no substantial differences from the main analysis (Figure 2). Our sensitivity analyses also examined the effect of varying the infection multiplier. Estimates based on multipliers of 3.4 and 4.7 differed in scale from those using the multiplier of 4 in the primary analysis but showed the same temporal patterns (Figure A4).

We also performed 7-day sliding window Rt estimation for the regions and counties. The results were similar to the state level results, and they were not shown here.

### 3.2. Policy Change Rt

**Figure 3:**
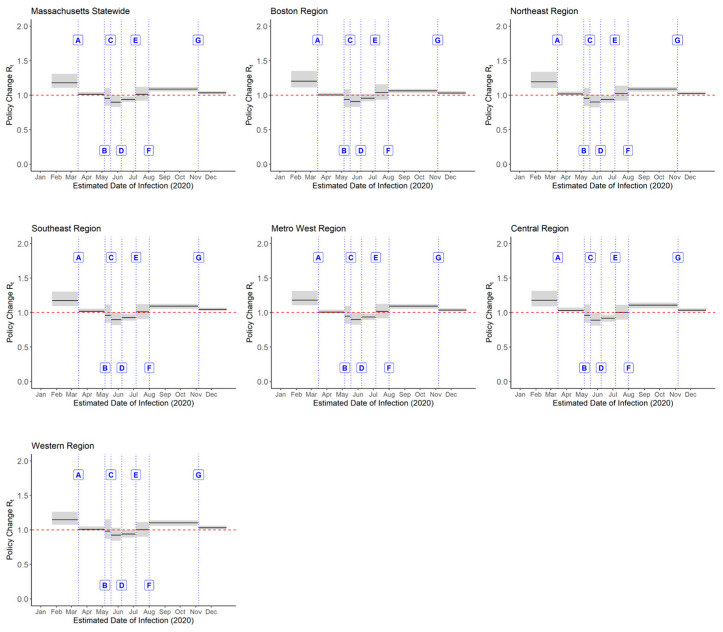
Policy change Rt estimates at the statewide and regional levels in Massachusetts in 2020. The black lines represent median Rt estimates, and the shaded areas represent the corresponding 95% CrI. Labels: A—School closure and gathering restrictions (15 March 2020); B—Mask mandate (6 May 2020); C—Phase 1 re-opening (18 May 2020); D—Phase 2 re-opening (8 June 2020); E—Phase 3 re-opening (6 July 2020); F—Travel and quarantine requirements (1 August 2020); G—Curfew and gathering restrictions (6 November 2020).

**Figure 4:**
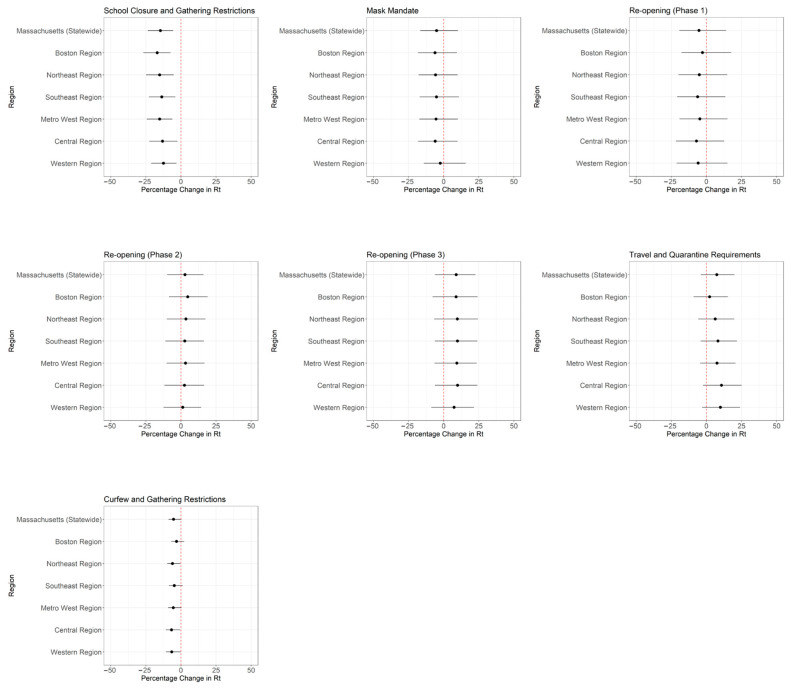
Median percentage change and corresponding 95% CrI of policy change Rt estimates at the statewide and regional levels in Massachusetts in 2020. The *x*-axis represents percentage change, with increases in Rt (positive values) shown to the right of the vertical dotted line at zero and decreases in Rt (negative values) shown to the left.

Throughout 2020, Massachusetts implemented a range of public health measures with differential effects on Rt across the state. Analysis of these changes (Figure 3 and Figure 4; Table A2) showed that statewide school closure and gathering restrictions (Policy A) were associated with a notable reduction in Rt (−14.7%; 95% CrI: −23.6%, −5.6%). Meanwhile, the mask mandate (Policy B), the re-opening phases (Policies C–E), travel and quarantine measures (Policy F), and curfew and gathering restrictions (Policy G) were not associated with statistically significant changes in Rt at the state level.

Regional analysis indicated variability in the effects of these NPIs (Figure 3 and Figure 4; Table A2). The Boston region experienced the largest reduction in Rt from school closure and gathering restrictions (−16.9%; 95% CrI: −26.9%, −7.5%), followed by the Northeast (−15.2%; 95% CrI: −24.9%, −5.1%) and Metro West (−15.2%; 95% CrI: −24.4%, −6.1%) regions. In contrast, the Western region showed no statistically significant reduction in Rt (−12.5%; 95% CrI: −21.3%, 3.2%). The mask mandate showed limited effect across all regions, which was consistent with state-level findings. Similarly, Phase 3 re-opening and travel and quarantine requirements were not associated with changes in Rt across regions. Curfew and gathering restrictions were only associated with reductions in Rt in the Central (−6.8%; 95% CrI: −10.8%, −0.5%) and Western (−6.6%; 95% CrI: −10.7%, −0.3%) regions.

**Figure A5:**
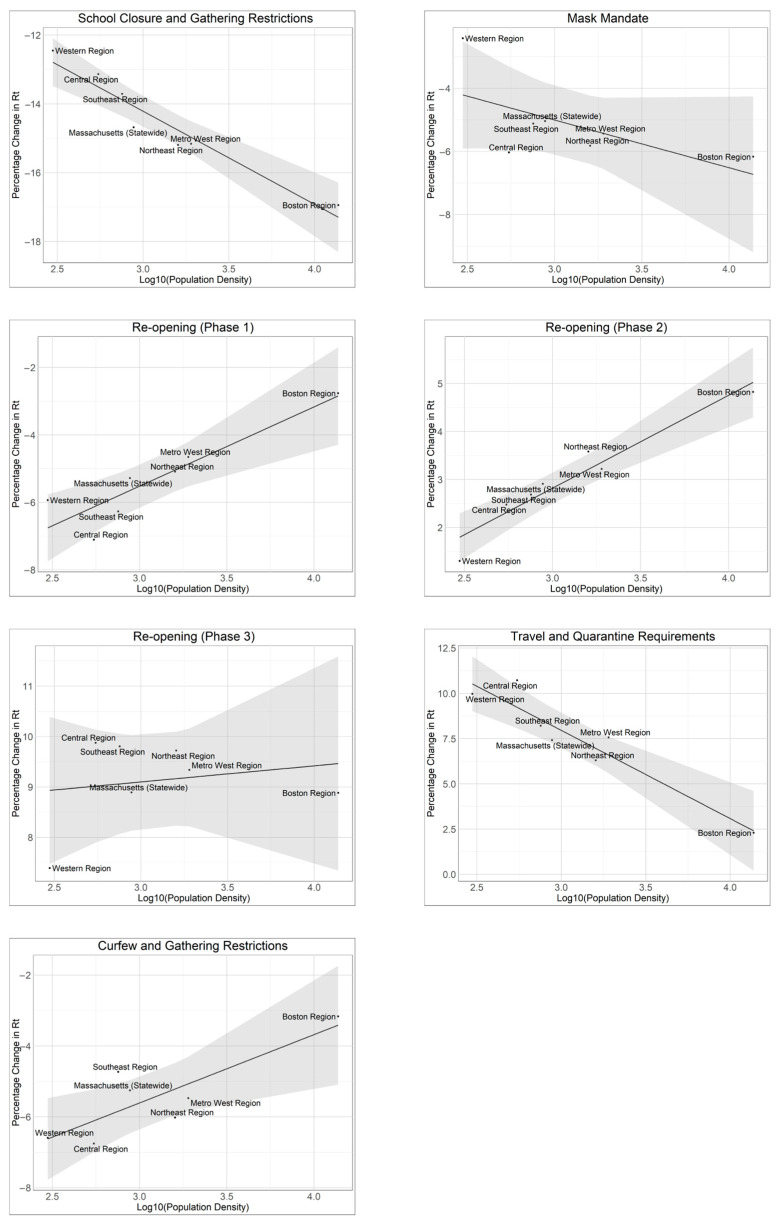
Linear regression plots of percentage change in Rt versus log_10_-transformed population density across Massachusetts regions for each major non-pharmaceutical intervention (NPI) implemented in 2020. Each panel displays a separate NPI, with regions labeled accordingly. Shaded areas represent 95% CrI.

We postulate that the magnitude of percentage change in Rt following policy changes is associated with regional population density (Table A3). Linear regression analyses demonstrated strong correlations between the implementation and relaxation of certain NPIs and log_10_-transformed population density. For example, for every 10-fold increase in population density, the percentage change in Rt decreased by 2.7% (95% CrI: −3.6%, −1.8%) following school closure and gathering restrictions, with an R^2^ of 0.93. In contrast, Phase 2 re-opening was associated with a 1.9% increase in the percentage change in Rt (95% CrI: 1.3%, 2.6%) per 10-fold increase in population density, also with an R^2^ of 0.93. Similarly, the implementation of travel and quarantine requirements in August 2020 was associated with a 4.9% decrease in the percentage change in Rt (95% CrI: −6.8%, −3.0%) per 10-fold increase in population density, with an R^2^ of 0.90. Other NPIs, such as the mask mandate and curfew restrictions, showed weaker correlations, as reflected by lower R^2^ values (Table A4; Figure A5).

**Figure A6:**
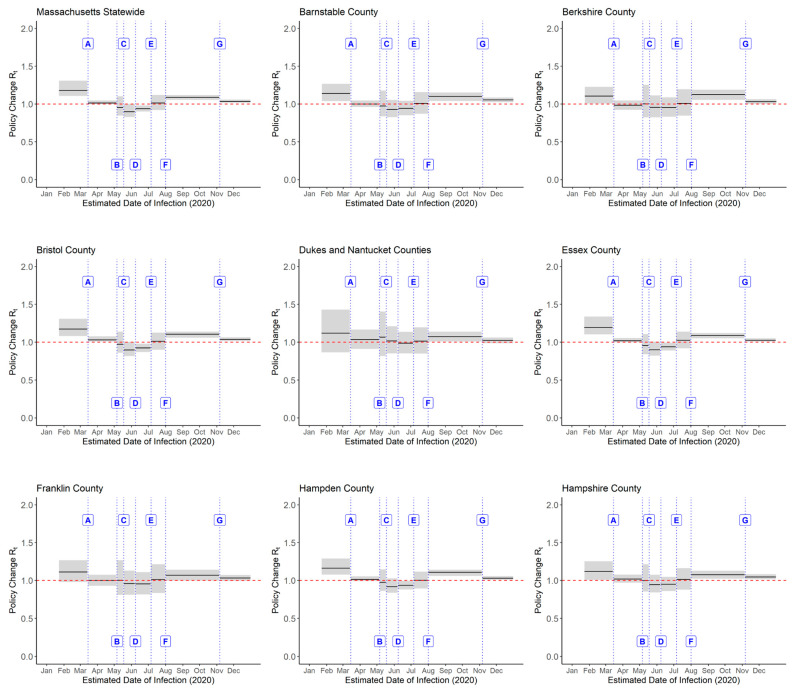

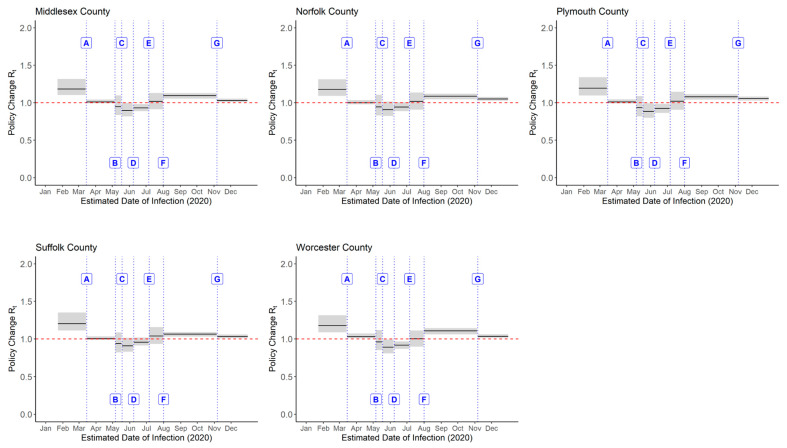
Policy change Rt estimates at statewide and county levels in Massachusetts in 2020. The black lines represent median Rt estimates, and the shaded areas represent the corresponding 95% CrI. Labels: A—School closure and gathering restrictions (15 March 2020); B—Mask mandate (6 May 2020); C—Phase 1 re-opening (18 May 2020); D—Phase 2 re-opening (8 June 2020); E—Phase 3 re-opening (6 July 2020); F—Travel and quarantine requirements (1 August 2020); G—Curfew and gathering restrictions (6 November 2020).

**Figure A7:**
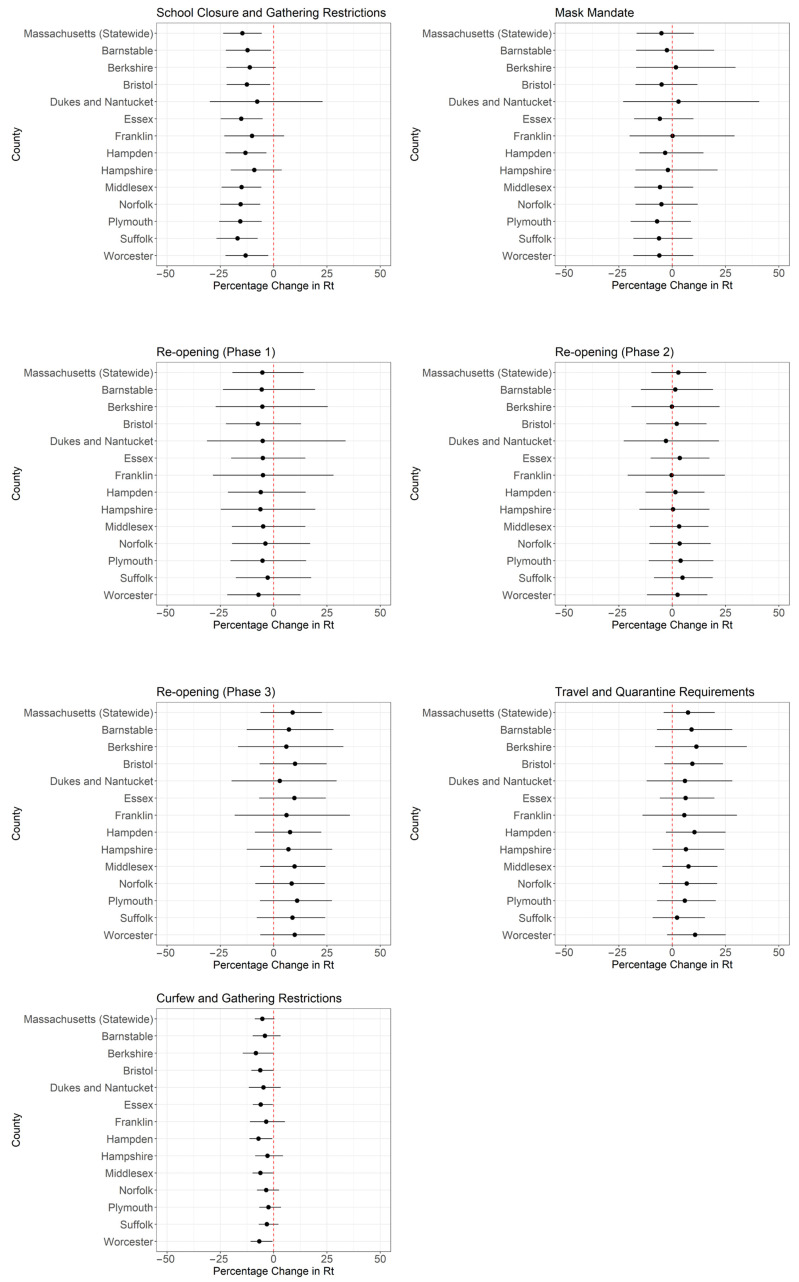
Median percentage change and corresponding 95% CrI of policy change Rt estimates at statewide and county levels in Massachusetts in 2020. The *x*-axis represents percentage change, with increases in Rt (positive values) shown to the right of the vertical dotted line at zero and decreases in Rt (negative values) shown to the left.

At the county level, both the median Rt estimates (Figure A6) and the percentage changes in Rt (Figure A7) were broadly consistent with the regional results, supporting the robustness of our findings. However, credible intervals were generally wider in counties with smaller populations.

## 4. Discussion

In this study, we estimated COVID-19 Rt in Massachusetts from January 2020 to July 2022 and examined how NPIs were associated with the transmission dynamics of SARS-CoV-2 in Massachusetts during 2020. Specifically, we assessed the association between seven major statewide NPI policy changes in 2020 and SARS-CoV-2 transmission across six regions of the state (Figure 4). Our findings indicate that early interventions, such as school closure and gathering restrictions, were associated with reductions in Rt. In contrast, none of the subsequent NPI policy implementation and relaxation was associated with statistically significant changes in Rt at the state level, and the magnitude of percentage change in Rt varied by region. Notably, the mask mandate introduced in May 2020 was not associated with a significant change in Rt. This may be due to the short interval (less than two weeks) between the implementation of the mask mandate (Policy B) and Phase 1 re-opening (Policy C), which could have confounded the observed trends. Prior research by Li et al. suggested the effects of NPIs may not be immediate, with their maximal effect potentially delayed by up to four weeks, and that the optimal timing of their effect varies by intervention [33].

Our findings on the effects of widely implemented NPIs in reducing COVID-19 Rt align with similar studies conducted in 13 other U.S. states and three Canadian provinces [6,7,8,9,10,11,34]. In Kentucky, North Dakota, Alberta, British Columbia, and Ontario, the implementation of school closure early in the pandemic had a significant impact on reducing transmission, with reductions in Rt ranging from −18.0% to −42.6%. Similarly, other physical distancing measures, including business closures and stay-at-home orders, were associated with reductions in Rt ranging from −20.0% to −62.7%. Although some states implemented multiple NPIs concurrently, potentially compounding their effects, the findings consistently highlight the effectiveness of early interventions, such as school closure and physical distancing, in controlling transmission before vaccines were available. K–12 school closure, in particular, have proven effective in mitigating influenza pandemics by reducing case burden [35,36]. Our findings, along with the aforementioned studies, provide further evidence that limiting physical gatherings may reduce COVID-19 transmission. These findings are also consistent with those of Auger et al., who reported that statewide school closure was temporally associated with reductions in COVID-19 incidence and mortality [37]. This underscores the role of children in transmission, likely due to prolonged close contact and mild or asymptomatic presentations [38]. While the effectiveness of school closure in reducing transmission is well-documented, the negative consequences of prolonged closures on children’s education have also been emphasized [39,40]. Decisions regarding school closure should therefore be made with careful consideration to balance educational needs with public health goals. Furthermore, we acknowledge the negative mental health effects of prolonged social distancing measures, which may be associated with the observed increase in opioid overdose events and overdose-related deaths in the U.S. at the onset of the COVID-19 pandemic [41,42]. Decision-makers, therefore, must carefully weigh the benefits and drawbacks of social distancing measures when responding to future pandemics caused by emerging respiratory pathogens.

Our regional analysis showed variability in policy change Rt across the six regions of Massachusetts. Following school closure and gathering restrictions in March 2020, the Boston, Northeast, and Metro West regions experienced slightly larger reductions in Rt (−15.2% to −16.9%) compared to the statewide average (−14.7%), while the Southeast, Central, and Western regions saw smaller reductions (−12.5% to −13.7%). However, the CrIs for these estimates overlapped considerably (Table A4), suggesting that there were no statistically significant differences in Rt reductions between regions. Given the similarity in Rt changes across regions, these differences are unlikely to warrant tailored interventions at the regional level.

The mask mandate showed limited effects across all regions, with reductions in Rt ranging from −2.4% to −6.6%. However, as multiple NPIs were implemented around the same time, it is difficult to isolate the specific impact of the mask mandate. Given that Rt had already been driven low by school closure and gathering restrictions since 15 March 2020, the marginal effect of adding a mask mandate to the existing package of NPIs on 6 May 2020, in further reducing Rt was small. This does not imply that masks themselves were ineffective. Rather, the population-level effectiveness of masking depends on adherence. Evidence from U.S. states indicates that those with ≥75% adherence, including Massachusetts, experienced substantially lower COVID-19 incidence in subsequent months, whereas states with lower adherence saw much higher case rates [43]. In this context, the statewide mask mandate was likely more important for encouraging protective behaviors that reduced individual risk of infection and for preparing the public for Phase 1 reopening than for producing an immediate reduction in Rt at the population level. Subsequently, Phase 2 and Phase 3 re-openings were associated with slight increases in Rt in several regions. Given the uncertainty around Rt estimates, the percentage change in Rt was also highly uncertain and statistically insignificant. Taken together, while interventions like school closure and gathering restrictions were associated with reductions in Rt, other measures such as the mask mandate and re-opening phases did not show statistically significant effects across regions. These findings indicate that not all NPIs were equally effective in reducing transmission at the population level, and their marginal effects were difficult to detect in real-world settings given the observational nature of the study design.

While our analysis did not find statistically significant associations between certain NPIs, such as the mask mandate and travel and quarantine requirements, and reductions in Rt, a key consideration in pandemic emergency response is that proactive measures, even with modest effects, are often preferable to inaction. In the context of a rapidly spreading pandemic before vaccines became available, even small reductions in spread could contribute to improved health outcomes at the population level. These effects might be amplified when multiple interventions were implemented in combination, potentially leading to greater overall reductions in transmission [44]. Furthermore, these interventions, when coupled with effective public health messaging that fostered compliance and signaled government action to curb transmission, could reduce strain on healthcare systems and lower the risk of more severe outcomes [45]. Future research could examine the effects of population immunity over time on transmission dynamics and investigate factors contributing to variation in intervention outcomes across regions. These efforts could strengthen pandemic preparedness and inform more effective, evidence-based public health responses.

### Limitations

This study was subject to several limitations. First, irregularities in public health surveillance reporting, such as negative or zero incident case counts, necessitated data imputation using surrounding values. While this approach might not fully reflect true case counts, it provided reasonable estimates that reduced discontinuities caused by reporting anomalies and enabled a more interpretable dataset for Rt analysis. Second, our deconvolution process to estimate infection time relied on the *incidental* package’s default infection-to-report time lag distribution, which was derived from a 2020 Florida dataset. However, the time lag distribution in Massachusetts could differ from that in Florida, and this distribution might change over time. Third, the underreporting of COVID-19 cases, particularly asymptomatic or mildly symptomatic infections, might have introduced bias into infection estimates. To address this, we applied a Poisson-distributed multiplier of 4 to estimate total infections [6,18]. While this provided a simple adjustment for underreporting, it did not account for regional or temporal variation in reporting rates or symptom presentation. These differences likely affected the accuracy of infection estimates and might limit the comparability of results across regions and over time. As such, findings related to spatial or temporal differences in transmission should be interpreted with caution. Fourth, Rt was estimated using the *EpiEstim* package, which assumes a fixed serial interval distribution. While serial intervals might have changed over time due to shifting NPIs or behavioral adaptations [46], time-varying data on serial intervals were not available for most of the study period. As such, the use of a constant serial interval was considered a reasonable approximation, though it may introduce bias during periods of rapid transmission or major policy change. Fifth, the policy change Rt analysis was segmented into seven non-overlapping time windows defined by key Executive Orders related to NPIs. This segmentation inevitably involved some arbitrariness, but it offered a systematic way to examine the association between policy changes and transmission dynamics. Because public adherence and enforcement did not always coincide with the exact dates of policy enactment, the analysis could not fully capture the timing of behavioral shifts. A policy change Rt estimate between the time points of two major policy enactments was essentially an average of Rt estimates over that period of time, reflecting the average transmission potential over the time period under a specific combination of policy mandates. That said, anchoring estimates to official enactment dates could smooth short-term fluctuations and capture the broader temporal effects of major interventions. Sixth, this is an ecological study using aggregate data. Association at the group level may not reflect the relationship at the individual level. Ecological fallacy is a possibility. That said, this is an appropriate study design, given our research questions.

## 5. Conclusions

The Massachusetts COVID-19 Rt case study provides valuable insights into the impact of public health interventions during the early stages of the pandemic. While modest regional differences in Rt were observed, overlapping CrIs suggest that these differences were not statistically significant enough to warrant region-specific interventions. Our findings highlight the importance of early and broad measures, such as school closure and gathering restrictions, in mitigating transmission.

## Figures and Tables

**Table 1:** Major policy measures enacted in Massachusetts in response to the COVID-19 pandemic, covering the period from April through November 2020.

Label	Start Date	Executive Order	Policy Declaration	Details	End Date
A	15 March 2020	Executive Order no. 3 [22], Executive Order no. 5 [23], Executive Order no. 13 [24].	School closure and gathering restrictions	All public and private schools were closed. Dining at restaurants was prohibited, and gatherings of more than 25 people were banned. A stay-at-home advisory was issued. Nonessential businesses were closed; restaurants were limited to takeout and delivery services; and gatherings of more than 10 people were prohibited.	Executive Order no. 3 remained in effect until 6 April 2020. Executive Order no. 5 was rescinded by Order no. 13 effective 23 March 2020. Executive Order no. 13 was rescinded by Order no. 37 effective 6 June 2020.
B	6 May 2020	Executive Order no. 31 [25].	Mask mandate	Face coverings were required in public.	Rescinded by Order no. 55 effective 6 November 2020.
C	18 May 2020	Executive Order no. 33 [26].	Re-opening (Phase 1)	A re-opening plan was released, and the stay-at-home advisory was renamed the “Safer at Home” advisory. The plan allowed places of worship, essential businesses, manufacturing facilities, and construction sites to re-open under strict safety guidelines. Hospitals and health centers were also permitted to re-open for urgent preventive care and treatment services for high-risk patients.	Rescinded by Order no. 69 effective 28 May 2021.
D	8 June 2020	Executive Order no. 37 [27].	Re-opening (Phase 2)	Massachusetts entered Phase Two of the re-opening plan.	Rescinded by Order no. 69 effective 28 May 2021.
E	6 July 2020	Executive Order no. 43 [28].	Re-opening (Phase 3)	Massachusetts entered Phase Three of the re-opening plan.	Rescinded by Order no. 69 effective 28 May 2021.
F	1 August 2020	Executive Order no. 45 [29].	Travel and quarantine requirements	New travel and quarantine requirements for visitors and returning residents were announced. Travelers and residents returning from out of state were required to quarantine for 14 days unless arriving from an exempt state or with proof of a negative COVID-19 test within the past 72 h.	Rescinded by COVID-19 Order no. 66 effective 22 March 2021.
G	6 November 2020	Executive Order no. 53 [30], Executive Order no. 54 [31], Executive Order no. 55 [32].	Curfew and gathering restrictions	A statewide curfew for businesses, stricter limits on indoor gatherings, and an expanded face mask mandate were introduced. The curfew required businesses such as theaters and casinos to close by 9:30 p.m., and restaurants to stop providing table service at the same time. Indoor gatherings at private homes were limited to 10 people, and outdoor gatherings to 25. All social events, whether indoors or outdoors, were also required to end by 9:30 p.m. Face coverings were required in all public settings for individuals over the age of five, even when social distancing could be maintained.	Executive Order no. 53 was rescinded by Order no. 69 effective 28 May 2021. Executive Order no. 54 was superseded by Order no. 57 effective 13 December 2020. Executive Order no. 55 was rescinded by Order no. 67 effective 30 April 2021.

## Data Availability

COVID-19 data analyzed in this project is publicly available at the COVID-19 Data Repository by the Center for Systems Science and Engineering (CSSE) at Johns Hopkins University. The original contributions presented in this study are included in the article/Supplementary Material. Further inquiries can be directed to the corresponding author.

## Supplementary Materials

The following supporting information can be downloaded at: https://www.mdpi.com/article/10.3390/epidemiologia7010015/s1, File S1 (an RMarkdown file) includes the code used for data analysis and visualization; and File S2 (an R file) contains the custom R functions used in the analysis.

## Abbreviations

The following abbreviations are used in this manuscript:

CORI	Criminal Offender Record Information
COVID-19	Coronavirus disease 2019
CrI	Credible intervals
CSSE	Center for Systems Science and Engineering
DPH	Department of Public Health
NPI	Nonpharmaceutical interventions
RMV	Registry of Motor Vehicles
Rt	Time-varying reproduction number
SARS-CoV-2	Severe acute respiratory syndrome coronavirus 2

## Appendix A

**Table A1:** A chronological list of policy measures enacted in Massachusetts in response to the COVID-19 pandemic from March to December 2020.

Issued Date	Type of Order	Order	Rescinded
11 March 2020	Health Care Workforce	Board of Registration in Nursing Policy expedited the processing of reciprocal license applications for nurses licensed in another jurisdiction to allow one business day processing [47].	
12 March 2020	Congregate Care	Massachusetts Department of Public Health (DPH) Order restricting visitors to nursing and rest homes [48].	
12 March 2020	Miscellaneous/Other	Governor’s COVID-19 Order no. 1 suspended certain provisions of the Open Meeting Law [49].	Rescinded 15 June, per Order no. 69
13 March 2020	Limits on Gatherings	Governor’s COVID-19 Order no. 2 prohibited gatherings of more than 250 people [50].	Rescinded by Order no. 5
15 March 2020	K-12 Schools	Governor’s COVID-19 Order no. 3 temporarily closed K-12 schools until 6 April [22].	
15 March 2020	Health Insurance	Governor’s COVID-19 Order no. 4 required insurers to cover all medically necessary telehealth services, reimbursed at the same rate as in-person services, without cost-sharing [51].	Rescinded by Order no. 61, Rescinded by Order no. 69
15 March 2020	Limits on Gatherings	Governor’s COVID-19 Order no. 5 prohibited gatherings of more than 25 people [23].	Rescinded by Order no. 13
15 March 2020	Registry of Motor Vehicles	Governor’s COVID-19 Order no. 6 authorized the Registry of Motor Vehicles (RMV) to temporarily extend licenses, permits, and other ID cards [52].	Rescinded by Order no. 11
15 March 2020	Health Care Delivery	DPH Order regarding non-essential, elective invasive procedures in hospitals and ambulatory surgical centers [53].	
15 March 2020	Labs and Hospitals	DPH Order regarding hospital visitor restrictions [54].	
15 March 2020	Congregate Care	DPH Order restricting visitors at assisted living facilities [55].	
17 March 2020	Health Care Workforce	Governor’s COVID-19 Order no. 7 expanded access to physician services [56].	Rescinded by Order no. 69
17 March 2020	Health Care Workforce	Governor’s COVID-19 Order no. 8 extended registration of certain licensed healthcare professionals [57].	Rescinded by Order no. 69
17 March 2020	Health Care Workforce	DPH Order implementing emergency credentialing and licensed staff transfer procedures for medical facilities in the Commonwealth [58].	
17 March 2020	Health Care Delivery	DPH Order regarding Emergency Medical Services care [59].	
18 March 2020	State Permits, Licenses and Inspections	Governor’s COVID-19 Order no. 9 extended the registrations of certain licensed professionals [60].	Rescinded by Order no. 41
18 March 2020	Early Education and Child Care	Governor’s COVID-19 Order no. 10 closed child care programs and authorized the temporary creation of emergency child care programs [61].	
18 March 2020	Health Care Workforce	DPH Order regarding the sharing of critical information with first responders [62].	
18 March 2020	Health Care Workforce	DPH Order regarding the flexible reassignment of Physician Assistants [63].	
18 March 2020	Pharmacy and Grocery Operations	DPH Order regarding the administration of certain medications for the treatment of opioid misuse disorder [64].	
20 March 2020	Registry of Motor Vehicles	Governor’s COVID-19 Order no. 11 first authorized actions to limit in-person transactions at the RMV [65].	Rescinded by Order no. 69
20 March 2020	State Permits, Licenses and Inspections	Governor’s COVID-19 Order no. 12 permitted the temporary conditional deferral of certain inspections of residential real estate [66].	Rescinded by Order no. 41
23 March 2020	Limits on Gatherings	Governor’s COVID-19 Order no. 13 prohibited gatherings of more than 10 people until 7 April [24].	Rescinded by Order no. 37
23 March 2020	Essential Services	Governor’s COVID-19 Order no. 13 assured the continued operation of essential services and closed certain workplaces [24].	Rescinded by Order no. 37
23 March 2020	Governor’s Council	Governor’s COVID-19 Order no. 14 allowed for remote participation for the Governor’s Council [67].	Rescinded by Order no. 29
24 March 2020	Health Care Delivery	DPH Order exempting certain activities necessary to address COVID-19 from determination of need approval [68].	
24 March 2020	Pharmacy and Grocery Operations	DPH Order regarding modifications to pharmacy practice [69].	
25 March 2020	Early Education and Child Care	Governor’s COVID-19 Order no. 15 extended the closure of non-emergency child care programs [70].	
25 March 2020	K-12 Schools	Governor’s COVID-19 Order no. 16 extended the closure of K-12 schools until 4 May [71].	
26 March 2020	State Permits, Licenses and Inspections	Governor’s COVID-19 Order no. 17 suspended state permitting deadlines and extended the validity of state permits [72].	Rescinded by Order no. 42
26 March 2020	State Permits, Licenses and Inspections	Governor’s COVID-19 Order no. 18 extended certain professional licenses, permits, and regulations issued by Commonwealth agencies [73].	Rescinded by Order no. 41
26 March 2020	Health Care Workforce	DPH Order addressing independent prescriptive practice for advanced practice registered nurses [74].	
29 March 2020	Health Care Workforce	DPH Order authorizing DPH’s Office of Preparedness and Emergency Management to conduct Criminal Offender Record Information (CORI) checks on volunteers without a notarized CORI Acknowledgement Form in response to the COVID-19 emergency [75].	
29 March 2020	Congregate Care	DPH Order regarding long-term care facility resident transfers and discharges [76].	
30 March 2020	Miscellaneous/Other	Governor’s COVID-19 Order no. 19 regarding the conduct of stakeholder meetings by public companies [77].	Rescinded by Order no. 41
30 March 2020	Miscellaneous/Other	Governor’s COVID-19 Order no. 20 authorized EOHSS to adjust essential provider rates [78].	Rescinded by Order no. 41
31 March 2020	Limits on Gatherings	Governor’s COVID-19 Order no. 21 extended the prohibition on gatherings of more than 10 people until 4 May [79].	Rescinded by Order no. 30
31 March 2020	Essential Services	Governor’s COVID-19 Order no. 21 extended the closure of certain workplaces until 4 May [79].	Rescinded by Order no. 30
1 April 2020	Congregate Care	DPH Order regarding administration of certain medications in Community Programs [80].	
2 April 2020	Recreation and Beaches	Governor’s COVID-19 Order no. 22 limited access to and use of state beaches [81].	Rescinded by Order no. 34
2 April 2020	Congregate Care	DPH Order regarding the provision of skilled nursing care in Assisted Living Residences [82].	
3 April 2020	Health Care Workforce	DPH Order regarding expanded practice by advanced practice registered nurses at Department of Mental Health facilities [83].	
3 April 2020	Health Care Workforce	DPH Order expanding the availability of certain healthcare providers, replacing the previous 29 March Order [84].	
3 April 2020	Pharmacy and Grocery Operations	DPH Order permitted licensed pharmacies to create and sell hand sanitizer over the counter, replacing a previous 15 March Order [85].	
4 April 2020	Pharmacy and Grocery Operations	DPH Order regarding the COVID Pharmacy Assistance Team [86].	
6 April 2020	Health Care Delivery	DPH Order regarding submission of written follow-up procedures for oral prescriptions [87].	
7 April 2020	Pharmacy and Grocery Operations	DPH Order addressed the operation of grocery stores and pharmacies [88].	Rescinded by DPH Order of 10 July
8 April 2020	Labs and Hospitals	DPH Order regarding collection of complete demographic information on patients with confirmed or suspected COVID-19 [89].	
9 April 2020	Health Care Workforce	Governor’s COVID-19 Order no. 23 provided accelerated licensing of physicians educated in foreign medical schools [90].	Rescinded by COVID-19 Order no. 41, but licenses issued remain valid for 2 years from the date of issuance.
9 April 2020	Health Care Workforce	Governor’s COVID-19 Order no. 24 authorized nursing practice by nursing school graduates and students in their final semester of nursing education programs [91].	Rescinded by Order no. 69
9 April 2020	Health Insurance	Governor’s COVID-19 Order no. 25 mandated that insurers cover all medically required emergency and inpatient services for COVID-19 treatment without any cost-sharing [92].	Rescinded by Order no. 61, Rescinded by Order no. 69
16 April 2020	Early Education and Child Care	Governor’s COVID-19 Order no. 26 authorized the creation and operation of emergency residential programs and emergency placement agencies for children [93].	Rescinded by Order no. 69
20 April 2020	Health Care Delivery	DPH Order regarding crisis standards of care [94].	Rescinded by DPH Order issued 19 June 2020
21 April 2020	Early Education and Child Care	Governor’s COVID-19 Order no. 27 extended the closure of non-emergency child care programs [95].	
21 April 2020	K-12 Schools	Governor’s COVID-19 Order no. 28 extended the closure of K-12 schools until 29 June [96].	
28 April 2020	Governor’s Council	Governor’s COVID-19 Order no. 29 revised the order allowing for remote participation for the Governor’s Council [97].	Rescinded by Order no. 69
28 April 2020	Limits on Gatherings	Governor’s COVID-19 Order no. 30 extended the prohibition on gatherings of more than 10 people until 18 May [98].	Rescinded by Order no. 38
28 April 2020	Essential Services	Governor’s COVID-19 Order no. 30 further extended the closure of certain workplaces until 18 May [98].	Rescinded by Order no. 32
28 April 2020	Health Care Workforce	DPH Order regarding staffing ratios in out-of-hospital dialysis units [99].	
28 April 2020	Labs and Hospitals	DPH Order allowing certain referrals to clinical laboratories for COVID-19 testing [100].	
28 April 2020	Miscellaneous/Other	DPH Guidance related to the operation of nurseries, greenhouses, garden centers, and agricultural supply stores, replacing previous 4 April Guidance [101].	
28 April 2020	Miscellaneous/Other	DPH Guidance regarding hotels and motels [102].	
1 May 2020	Masks and Face-Coverings	Governor’s COVID-19 Order no. 31 required face-coverings in public places where social distancing was not possible and at all times on public transit and in retail settings [25].	Rescinded by Order no. 55 effective 6 November 2020
15 May 2020	Essential Services	Governor’s COVID-19 Order no. 32 temporarily extended COVID-19 Order no. 13 for 24 additional hours ahead of the release of the reopening plan [103].	Rescinded by Order no. 33
18 May 2020	Reopening Protocols and Business Restrictions	Governor’s COVID-19 Order no. 33 implemented phased reopening of workplaces, imposed workplace safety measures to address COVID-19, and authorized reopening of Phase I enterprises [26].	Rescinded by Order no. 69
18 May 2020	Recreation and Beaches	Governor’s COVID-19 Order no. 34 expanded access to and use of state beaches and addressed other outdoor recreational activities [104].	Rescinded by Order no. 69
18 May 2020	Health Care Workforce	DPH Order exempting certain hospitals from nurse staffing requirements of M.G.L. c. 111, § 231 [105].	Rescinded 1 March 2021
1 June 2020	Reopening Protocols and Business Restrictions	Governor’s COVID-19 Order no. 35 clarified the progression of the Commonwealth’s phased reopening plan and authorized certain reopening preparations at Phase II workplaces [106].	Rescinded by Order no. 69
1 June 2020	Early Education and Child Care	Governor’s COVID-19 Order no. 36 authorized preparations for the reopening of child care programs, and for the issuance of minimum health and safety standards for child care [107].	Rescinded 15 June, per Order no. 69
6 June 2020	Reopening Protocols and Business Restrictions	Governor’s COVID-19 Order no. 37 authorized reopening of Phase II enterprises (Step 1) [27].	Rescinded by Order no. 69
6 June 2020	Limits on Gatherings	Governor’s COVID-19 Order no. 38 extended the prohibition on gatherings of more than 10 people and clarified its application [108].	Rescinded by Order no. 44
12 June 2020	Registry of Motor Vehicles	Governor’s COVID-19 Order no. 39 s authorized actions to limit in-person transactions at the RMV [109].	Rescinded by Order no. 47
19 June 2020	Reopening Protocols and Business Restrictions	Governor’s COVID-19 Order no. 40 authorized reopening of Phase II enterprises (Step 2) [110].	Rescinded by Order no. 69
26 June 2020	Early Education and Child Care	Governor’s COVID-19 Order no. 41 authorized the reopening of child care programs and rescinded the authorization for emergency child care programs and 8 other orders [111].	Rescinded by Order no. 69
2 July 2020	State Permits, Licenses and Inspections	Governor’s COVID-19 Order no. 42 resumed state permitting deadlines and continued to extend the validity of certain state permits [112].	Rescinded 15 June, per Order no. 69
2 July 2020	Reopening Protocols and Business Restrictions	Governor’s COVID-19 Order no. 43 authorized reopening of Phase III enterprises (Step 1) [28].	Rescinded by Order no. 69
2 July 2020	Limits on Gatherings	Governor’s COVID-19 Order no. 44 raised the gathering limit to 25 persons indoors and 100 persons outdoors [113].	Rescinded by Order no. 46
2 July 2020	Congregate Care	DPH Order regarding provision of day program services provided in group settings, replacing previous 24 March Order [114].	
22 July 2020	Health Insurance	DPH Order prohibited billing uninsured individuals for COVID-19 testing [115].	
24 July 2020	Travel Restrictions	Governor’s COVID-19 Order no. 45 instituted a mandatory 14-day quarantine requirement for travelers arriving in Massachusetts [29].	Rescinded by COVID-19 Order no. 66 effective 22 March 2021
7 August 2020	Limits on Gatherings	Governor’s COVID-19 Order no. 46 revised gathering limits: reduced outdoor gatherings from 100 to 50, maintained indoor gathering limits at 25 [116].	Rescinded by Order no. 52
11 August 2020	Registry of Motor Vehicles	Governor’s COVID-19 Order no. 47 extended the second order authorizing actions to limit in-person transactions at the RMV [117].	Rescinded by Order no. 69
18 August 2020	Miscellaneous/Other	Governor’s COVID-19 Order no. 48 amended the administration of penalties issued pursuant to certain COVID-19 Orders [118].	Rescinded by Order no. 69
28 August 2020	Early Education and Child Care	Governor’s COVID-19 Order no. 49 authorized arrangements for child care and supervision during the school day to accommodate remote learning [119].	Rescinded 15 June, per Order no. 69
8 September 2020	Miscellaneous/Other	DPH Order related to the operation of farmers markets, farm stands, and CSAs, replacing previous 27 April Order [120].	Rescinded by DPH Order on 27 April 2021
10 September 2020	Reopening Protocols and Business Restrictions	Governor’s COVID-19 Order no. 50 made certain Phase III adjustments, including the extension of outdoor dining provisions and the opening of indoor and outdoor gaming arcades [121].	Section 1 rescinded 15 June, per Order no. 69
29 September 2020	Reopening Protocols and Business Restrictions	Governor’s COVID-19 Order no. 51 authorized reopening of Phase III enterprises (Step 2) in municipalities with reduced incidence of COVID-19 infection [122].	Superseded and rescinded by Order no. 56, then rescinded again by Order no. 69
29 September 2020	Limits on Gatherings	Governor’s COVID-19 Order no. 52 adjusted gathering limits: indoor gatherings at all venues limited to 25, outdoor gatherings at private residences limited to 50, and up to 100 people for outdoor gatherings in lower-risk communities [123].	Rescinded by Order no. 54
6 October 2020	Health Care Workforce	DPH Order regarding influenza vaccination for healthcare personnel at assisted living facilities [124].	
22 October 2020	Recreation and Beaches	DPH Order regarding the operation of indoor ice rinks and indoor ice skating and hockey facilities [125].	
2 November 2020	Reopening Protocols and Business Restrictions	Governor’s COVID-19 Order no. 53 required early closure of certain businesses and activities each night at 9:30 p.m. Special allowance for renewal of licenses for on-premises alcohol consumption remained in place [30].	Rescinded by Order no. 69
2 November 2020	Limits on Gatherings	Governor’s COVID-19 Order no. 54 reduced gathering size limits at private residences: 10 people indoors, 25 people outdoors. Also required all gatherings to end by 9:30 p.m and imposed a $500 fine for each person over the limit [31].	Superseded by Order no. 57 (13 December 2020)
2 November 2020	Masks and Face-Coverings	Governor’s COVID-19 Order no. 55 revised order requiring all persons to wear face-coverings in all public places, even where they are able to maintain 6 feet of distance. Allowed exceptions for residents with medical or disabling conditions, but employers and schools could require proof of such conditions [32].	Rescinded by Order no. 67 effective 30 April 2021
5 November 2020	Congregate Care	DPH Order regarding control of COVID-19 in long-term care facilities [126].	
6 November 2020	Reopening Protocols and Business Restrictions	Governor’s COVID-19 Order no. 56 revised provisions for Phase III reopening in municipalities with reduced incidence of COVID-19 infection [127].	Superseded and rescinded by Order no. 58, then rescinded by Order no. 69
7 December 2020	Health Care Delivery	DPH Order related to elective procedures [128].	Rescinded by DPH Order issued 1 March 2021
7 December 2020	Congregate Care	DPH Order related to COVID-19 testing in congregate care sites [129].	
8 December 2020	Limits on Gatherings	Governor’s COVID-19 Order no. 57 reduced the outdoor gathering limit statewide from 100 to 50 persons [130].	Sustained other limits from Order no. 54. (Adjusted by Order no. 59, and further adjustments made by Order no. 62, which rescinded Section 4.)
8 December 2020	Reopening Protocols and Business Restrictions	Governor’s COVID-19 Order no. 58 required return to Phase III, Step 1 reopening for all Massachusetts communities and made adjustments to Phase III, Step 1 protocols [131].	Rescinded and superseded by Order no. 65, then rescinded by Order no. 69
22 December 2020	Health Care Workforce	DPH Order regarding healthcare personnel influenza vaccination in long-term care facilities, adult day health programs, and out-of-hospital dialysis units, replacing the previous October 6 Order [132].	
22 December 2020	Limits on Gatherings	Governor’s COVID-19 Order no. 59 applied a limit of 10 persons for indoor gatherings, 25 persons for outdoor gatherings in private homes, event venues, and public spaces [133].	
22 December 2020	Reopening Protocols and Business Restrictions	Governor’s COVID-19 Order no. 59 applied a 25% capacity limit to businesses and activities across most sectors [133].	Rescinded by Order no. 69
23 December 2020	Health Care Workforce	DPH Order regarding healthcare personnel influenza vaccination in certain state-operated facilities [134].	

**Table A2:** Percentage changes in median Rt, with 95% credible intervals (CrI), by policy change for Massachusetts at statewide and regional levels.

Region	School Closure and Gathering Restrictions (%)	Mask Mandate (%)	Re-Opening (Phase 1) (%)	Re-Opening (Phase 2) (%)	Re-Opening (Phase 3) (%)	Travel and Quarantine Requirements (%)	Curfew and Gathering Restrictions (%)
Massachusetts statewide	−14.7 (−23.6, −5.6)	−5.0 (−16.7, 10.1)	−5.3 (−19.4, 14.0)	2.9 (−9.8, 16.0)	8.9 (−6.2, 22.6)	7.4 (−4.0, 19.9)	−5.3 (−8.9, 0.4)
Boston	−16.9 (−26.9, −7.5)	−6.2 (−18.2, 9.5)	−2.8 (−17.8, 17.6)	4.8 (−8.5, 19.0)	8.9 (−7.9, 24.2)	2.3 (−9.2, 15.3)	−3.2 (−6.9, 2.2)
Northeast	−15.2 (−24.9, −5.1)	−5.8 (−17.9, 10.0)	−5.1 (−19.9, 14.9)	3.6 (−10.1, 17.5)	9.7 (−6.8, 24.5)	6.3 (−5.8, 19.8)	−6.0 (−9.7, −0.4)
Southeast	−13.7 (−22.9, −4.0)	−5.1 (−17.1, 10.9)	−6.3 (−20.9, 13.5)	2.7 (−11.1, 16.3)	9.8 (−6.3, 24.1)	8.2 (−4.2, 21.8)	−4.7 (−8.6, 1.3)
Metro West	−15.2 (−24.4, −6.1)	−5.4 (−17.3, 10.0)	−4.7 (−19.2, 15.1)	3.2 (−10.3, 16.8)	9.3 (−6.6, 23.5)	7.6 (−4.5, 20.8)	−5.5 (−9.2, 0.4)
Central	−13.1 (−22.6, −2.5)	−6.0 (−18.3, 9.9)	−7.1 (−21.8, 12.6)	2.5 (−11.8, 16.5)	9.9 (−6.2, 24.0)	10.7 (−2.4, 25.1)	−6.8 (−10.8, −0.5)
Western	−12.5 (−21.3, −3.2)	−2.4 (−14.2, 15.7)	−5.9 (−21.3, 15.0)	1.3 (−12.4, 14.5)	7.4 (−8.8, 21.6)	10.0 (−3.0, 24.0)	−6.6 (−10.7, −0.3)

**Table A3:** Total population and population density (number of people per square mile) for each region in Massachusetts in 2020.

Region	Total Population	Population Density (People/mi^2^)
Boston	801,162	13,742.06
Northeast	787,038	1598.05
Southeast	1,324,045	754.36
Metro West	2,309,639	1902.50
Central	826,655	547.20
Western	824,464	297.61

**Table A4:** Summary data from a linear regression model examining the correlation between percentage change in Rt and log_10_-transformed population density across regions in Massachusetts.

Non-Pharmaceutical Interventions	Slope (95% Crl)	R^2^
School Closure and Gathering Restrictions	−2.7 (−3.6, −1.8)	0.93
Mask Mandate	−1.5 (−3.6, 0.6)	0.40
Re-opening (Phase 1)	2.3 (1.1, 3.6)	0.82
Re-opening (Phase 2)	1.9 (1.3, 2.6)	0.93
Re-opening (Phase 3)	0.3 (−1.5, 2.2)	0.04
Travel and Quarantine Requirements	−4.9 (−6.8, −3.0)	0.90
Curfew and Gathering Restrictions	1.9 (0.5, 3.4)	0.70
